# Active in situ and passive airborne fluorescence measurements for water stress detection on a fescue field

**DOI:** 10.1007/s11120-022-00983-y

**Published:** 2022-12-03

**Authors:** Ismael Moya, Hildo Loayza, María-Llanos López, Juan Manuel Sánchez, Yves Goulas, Abderrahmane Ounis, Roberto Quiroz, Alfonso Calera

**Affiliations:** 1 LMD/IPSL, CNRS, ENS, Ecole Polytechnique, Sorbonne Université, 91128 Palaiseau, France; 2 International Potato Center (CIP), Headquarters, P.O. Box 1558, Lima, Peru; 3 Innovation Department, AgriSat Iberia SL, 02006 Albacete, Spain; 4 Department of Applied Physics, Regional Development Institute (IDR), University of Castilla-La Mancha, 02071 Albacete, Spain; 5 CATIE-Centro Agronómico Tropical de Investigación Y Enseñanza, Cartago, Turrialba 30501 Costa Rica

**Keywords:** Chlorophyll fluorescence, Water stress, LED-induced fluorescence, Airborne measurements, Sun-induced fluorescence, Fescue

## Abstract

Ledflex is a fluorometer adapted to measure chlorophyll fluorescence at the canopy level. It has been described in detail by Moya et al. (2019), Photosynthesis Research. https://doi.org/10.1007/s11120-019-00642-9. We used this instrument to determine the effect of water stress on the fluorescence of a fescue field under extreme temperature and light conditions through a 12 days campaign during summer in a Mediterranean area. The fescue field formed part of a lysimeter station in "las Tiesas," near Albacete-Spain. In addition to the fluorescence data, the surface temperature was measured using infrared radiometers. Furthermore, "Airflex," a passive fluorometer measuring the filling-in of the atmospheric oxygen absorption band at 760 nm, was installed in an ultralight plane and flown during the most critical days of the campaign. We observed with the Ledflex fluorometer a considerable decrease of about 53% of the stationary chlorophyll fluorescence level at noon under water stress, which was well correlated with the surface temperature difference between the stressed and control plots. Airflex data also showed a decrease in far-red solar-induced fluorescence upon water stress in agreement with surface temperature data and active fluorescence measurements after correction for PS I contribution. Notwithstanding, the results from airborne remote sensing are not as precise as in situ active data.

## Introduction

Remote sensing is becoming a prerequisite for monitoring the photosynthetic vegetation state at the field level. Among available methods for plant studies, chlorophyll fluorescence plays an essential role as this emission is directly linked to photosynthesis. Light absorbed by plants between 350 and 750 nm ultimately leads to an excited state of chlorophyll *a* at the reaction center. Several ways of de-excitation are possible: photochemistry and the subsequent CO_2_ fixation are highly probable under favorable conditions, but the energy can also be dissipated as heat or emitted as fluorescence emission. Because these three deactivation pathways compete, fluorescence is highly variable, and its variations reflect the variations of the photosynthetic activity.

The efficiency of the fluorescence emission in vivo is very low (less than 1–2% of absorbed energy). Nevertheless, chlorophyll fluorescence is widely used in the laboratory as it is a specific emission of green plants. In addition, chlorophyll is probably one of the rare constituents of the biosphere to fluoresce in the red and far-red parts of the spectrum.

For the last 30 years, several modulated fluorometers have been proposed to the community. Among them, the Pulse Amplitude Modulation (PAM) fluorometer (Walz, Effeltricht, Germany) was the most popular (Schreiber et al. 1986). At the basis of this success was the "light-doubling" technique (Quick and Horton 1984), in which a faint (non-actinic) modulated light generates a synchronously detected fluorescence emission that changes when a continuous actinic light is superimposed. In addition, the possibility to saturate photosynthesis with intense sub-second light pulses allows for calculating several parameters like the effective yield of photosystem II photochemistry, the yield of non-photochemical energy dissipation, or the apparent electron transport rate. The drawback of these measurements is the need to work at the leaf level and near contact.

It is impossible to saturate the fluorescence by using large target sizes of > 1 m in diameter to integrate the spatial heterogeneity at the canopy level. One of the most accessible parameters is stationary fluorescence (Fs), as shown by Cerovic et al. (1996). Fs variations are much smaller in amplitude than the maximum fluorescence (Fm); however, obtaining qualitative but valuable information on the vegetation's physiological state by measuring Fs continuously from a fixed and constant position is possible. For example, one can consider the Fs/Fo ratio (Fo is the Fs signal measured during the night) to characterize water stress, as shown by Moya et al. (2019). In the present work, the authors used the same "Ledflex," a LED-based micro-LIDAR able to measure Fs continuously at several meters, in full sunlight, and over a target size of up to 1m^2^.

We decided to use this instrument during reversible water stress in a well-controlled fescue field of a lysimeter station in "las Tiesas," near Albacete-Spain. In addition to the fluorescence data, the surface temperature was measured using infrared radiometers (IRTs) (MI-210, Apogee Instruments Inc., Lugan, UT, USA). Last but not least, "Airflex," a passive fluorometer measuring the filling-in of the atmospheric oxygen absorption band (Moya et al. 2006; Rasher et al*.* 2009; Daumard et al. 2015), was installed in an ultralight plane and flown during the most critical days of the campaign.

The aims of this work were:

1. To test the capacity of stationary fluorescence at canopy scale (Fs) measured by Ledflex to discriminate between well-watered and water-stressed conditions. The case of a natural crop under full sunlight is of particular scientific interest.

2. To compare active Ledflex measurements of fluorescence changes with passive Airflex measurements of fluorescence changes.

3. To compare canopy temperature changes with Ledflex fluorescence changes.

## Material and methods

### Experimental area

**Fig. 1:**
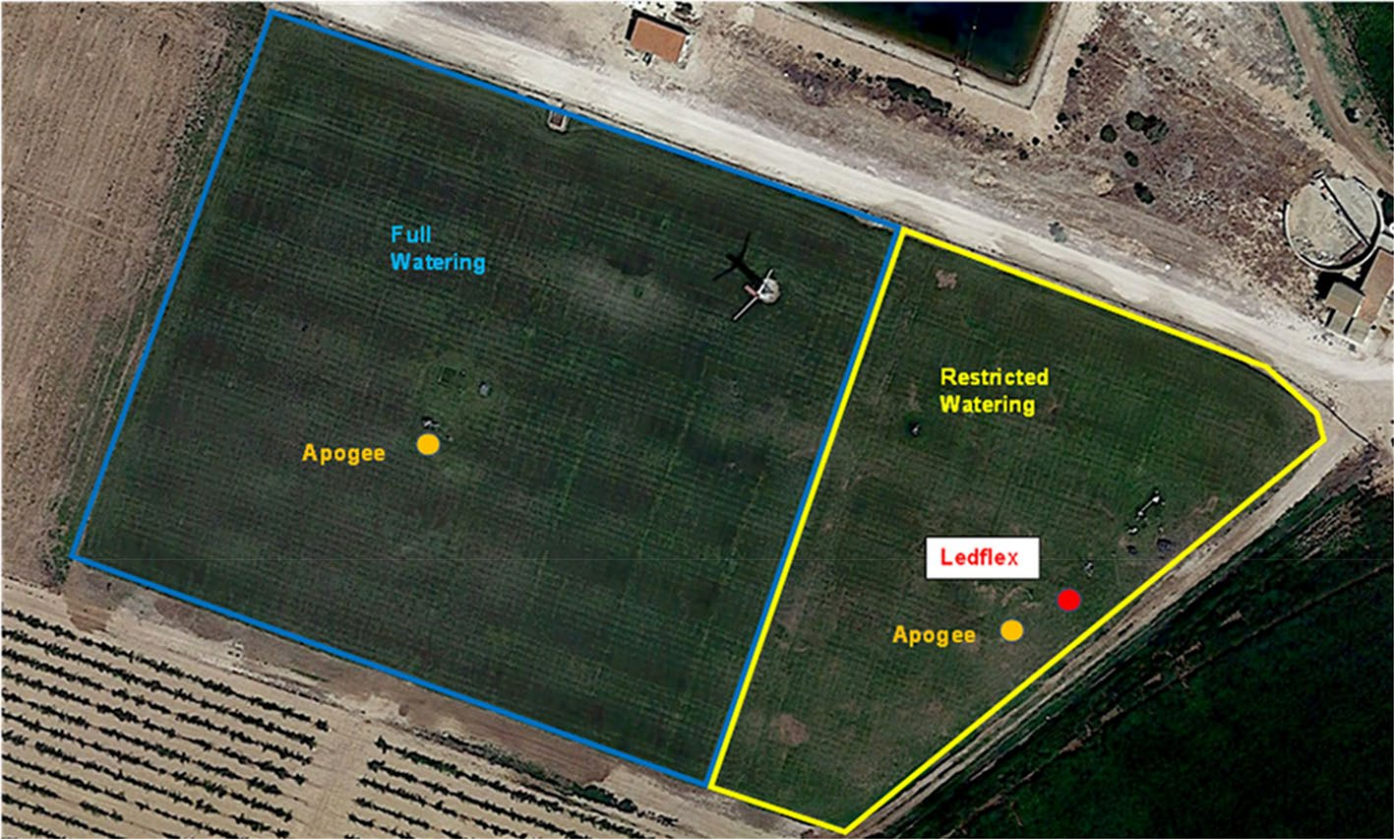
The fescue (*Festuca arundinacea Schreb*) meadowland. The plot was maintained in optimum growth conditions to measure reference evapotranspiration values (ETo) and has an automated sprinkler irrigation system of total underground coverage. The yellow line delineates the portion of the field where the irrigation was interrupted for several days to generate a controlled drought (stressed plot). (Figure modified from Google earth Pro V 7.3.4 (July 29, 2017). Las Tiesas, Barrax, Spain. 39° 03′ 37.45″ N, 2° 05′ 58.45″ W, Eye alt 901 m. July 01, 2022)

**Fig. 2:**
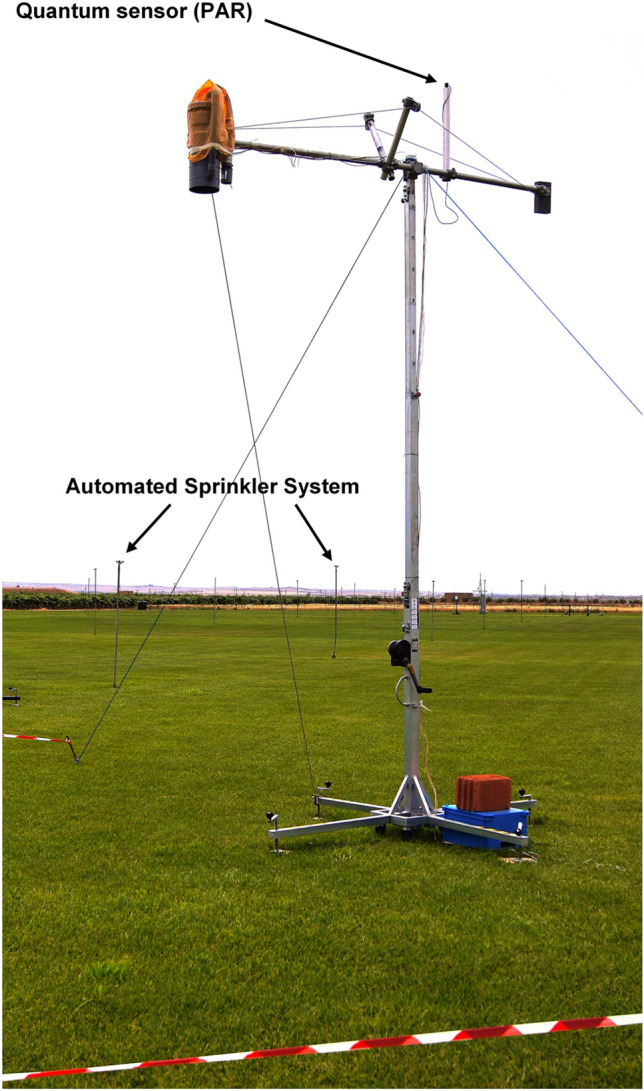
Ledflex in position over the fescue field

**Fig. 3:**
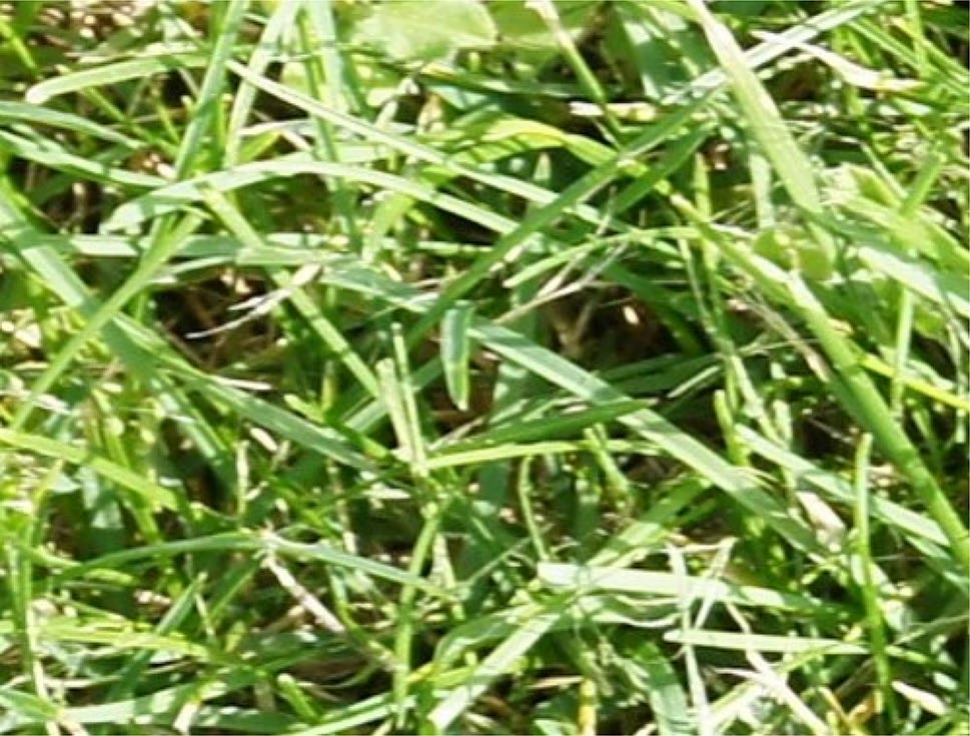
Detail of the fescue cover about 8–10 cm deep

The study was conducted in a flat country near Albacete in the South-East of Spain. The first measuring campaign in this zone was done in the summer of 2005 in the framework of the Earth Observation Envelope Programme of the European Space Agency (ESA) (Moya et al. 2006). The actual campaign lasted one month, from July 3 to August 2 during the summer of 2017. It occurred near Barrax (Albacete-Spain) in the experimental farm "las Tiesas," situated at 39.06º north, longitude 2.099º west, altitude 698 m. A lysimeter station was installed in the center of a ≈1-hectare plot of a fescue meadow (*Festuca arundinacea Schreb*), Fig. 1, maintained in optimum growth conditions with the object of measuring reference evapotranspiration (ETo) values (Fig. 2). The crop was kept between 0.08 and 0.12 m in height through weekly mowing (Fig. 3). The field was maintained in optimum growth conditions to measure reference evapotranspiration values and was irrigated regularly for three hours every two or three days from July 3rd until the end of the experiment by an automated sprinkler system of total underground coverage. Climatic data were recorded by the agro-meteorological station "Anchor station" thanks to an STA-212-PVC sonde. The agro-meteorological station was situated in the vicinity of the Ledflex instrument. In the actual campaign, the field was divided into two parts: i) A first part delineated by a blue line which was kept well-watered (Fig. 1) and ii) a second part delineated by a yellow line in Fig. 1 (hereafter named stressed plot) where the irrigation was interrupted for several days to generate a controlled drought (Fig. 3). The Ledflex fluorometer was installed in the stressed plot when it was still well-watered, as shown in Fig. 2. Irrigation of the stressed plot was interrupted from July 13 (last irrigation) until July 25, except for 1-h irrigation on July 21 and three hours on July 24. No rain occurred during this period. So, we assumed that on July 16, control conditions still prevailed for both plots and that maximum stress was obtained on July 24 in the stressed part.

An automated agrometeorological weather station (Anchor Station) was also used that provided 10-min, hourly, and daily recordings of the climatic data. In particular, the air temperature was measured at 0.5, 2, and 10 m above ground with an accuracy of $\pm$ 0.1 °C.

The original plan was to use a portable photosynthesis meter (LI-6400, Licor, USA) to gather gas exchange information to complement fluorescence measurements. However, this measurement was not taken because the fescue leaves were too small to fit the LI-6400 window. So instead, we used infrared thermometry (Apogee MI-210) in both parcels (control and stress) to continuously monitor surface temperatures day and night.

## LEDFLEX

This instrument has already been described by Moya et al. (2019). Briefly, Ledflex is a hardened fluorometer for continuous chlorophyll fluorescence measurements under natural illumination at distances up to 8 m. It has been designed to integrate the fluorescence emission of a target diameter of about 1 m. The light source consists of a group of pulsed blue Light Emitting Diodes (LEDs) (Thorlabs, Maisons-Laffitte, France) with peak emission at 470 nm and a full width at half maximum (FWHM) of 22 nm. The fluorescence emitted in response to the 5 μs width pulses of excitation light is separated from the reflected ambient light through a synchronized detection. The fluorescence signal is also spectrally selected with the combination of a high-pass filter (Schott RG665, 3 mm, λ > 665 nm, Edmund Optics, UK) and a low-pass filter (λ < 800 nm, Edmund Optics, UK) to reduce the detected spectral range to the functional zone (665–800 nm) where fluorescence is emitted. The target's reflected sunlight and LED-induced fluorescence were acquired simultaneously in the same field of view and the same spectral band. The incident photosynthetic active radiation (PAR) was acquired thanks to a quantum sensor (JYP 1000, SDEC-France, Tours, France) situated on a pole at the top of the Ledflex set-up. The Ledflex sensor was fixed on a vertical mast with a nadir viewing 4 m above ground (Fig. 2). The Ledflex sensor arm was directed south to avoid shadows in the target area. All the instruments were powered by a 12 V car battery recharged by a solar panel, allowing continuous measurements during night and day.

### Thermal-infrared radiometers

This experiment used two thermal-infrared radiometers (IRTs), Apogee MI-210 (Apogee Instruments, Inc.). These instruments have a broad thermal band (8–14 μm) with a field of view of 22º, a response time of 0.6 s, and an accuracy of ± 0.2 K. Calibration was assessed using a blackbody source (Hyperion R 982, Isotech, England). One of the IRTs was installed near Ledflex on the stressed plot. The second IRT was located in the "well-watered" control plot (see Fig. 1). The IRTs were fixed on a mast, at the height of 1.5 m above the ground, with a viewing angle of 45º, defining a surface target area of about 6.4 m^2^. Acquisition mode was set to perform a measurement every 30 s and then record an average every 30 min. This work did not apply atmospheric and emissivity corrections since only relative differences between well-watered and stressed crops were required.

The use of these broad-band IRTs is widespread in agronomic applications such as plant water status estimation or surface energy balance modeling (Sánchez et al. 2008, 2011, 2014, 2015, 2019) and also in surface temperature monitoring for remote sensing calibration/validation activities (Niclòs et al. 2015; Sobrino and Skoković 2016; Sánchez et al. 2020).

### Passive fluorescence measurements using Airflex

**Fig. 4:**
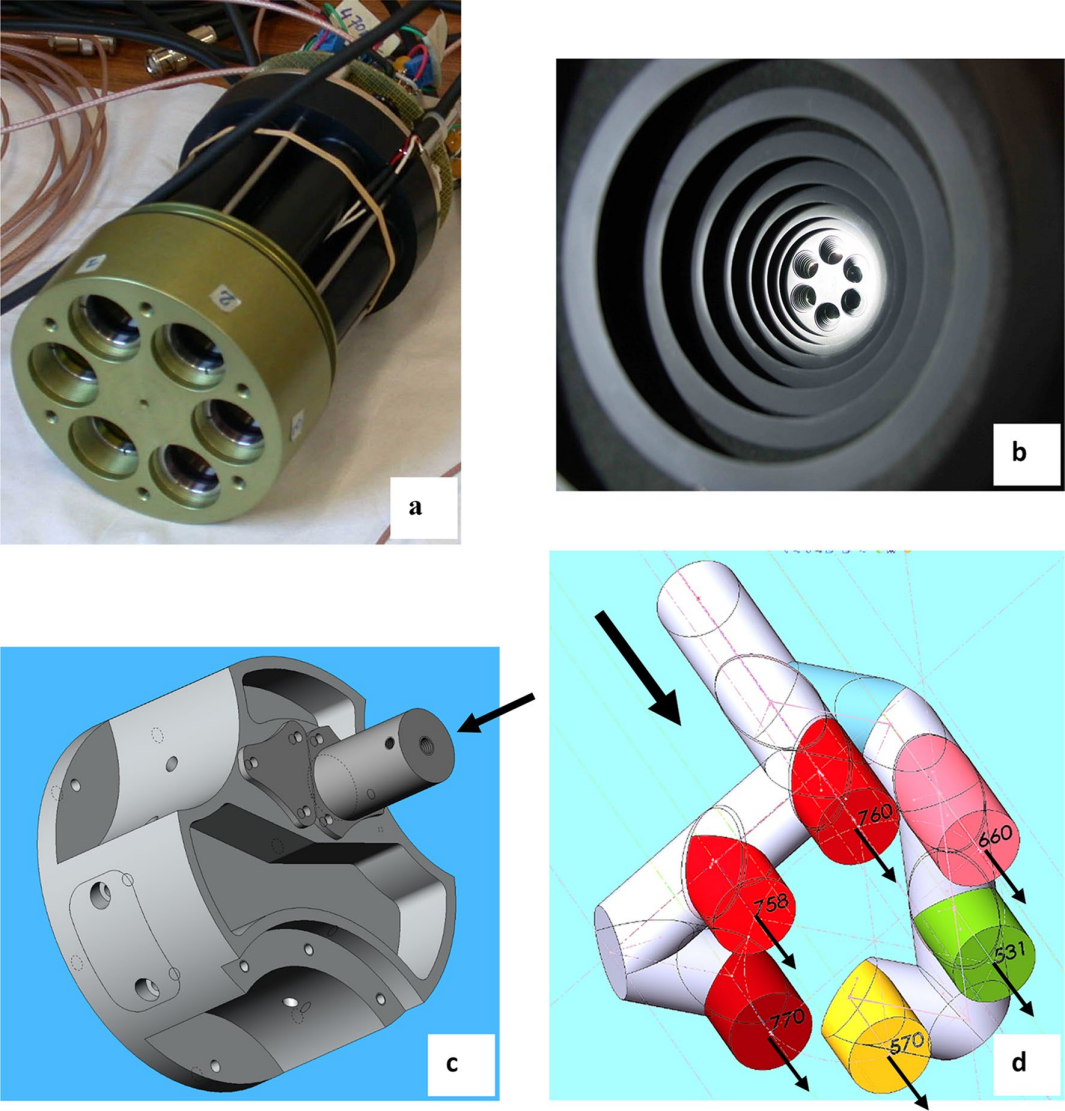
**a** Heart of the sensor, the objective exhibits six cavities containing the set of filters and the collimating lenses. The objective is maintained at 40 °C by a regulated heating system. **b** Hub. The objective is visible behind the baffle. A fiber optic replaced the black-painted baffle system's entrance to minimize stray light. **c** Beam splitter of the new version of Airflex. **d** Scheme showing the beam splitter interiors and how the beam collected by the optical fiber is divided into six channels thanks to a set of mirrors and dichroic filters

Airflex is an interference-filter-based airborne sensor developed in the Earth Observation Preparatory Program of the European Space Agency (ESA) framework and first described by Moya et al. (2006). See also Rascher et al. (2009) and Daumard et al. (2015). It is a 6-channels photoradiometer that measures the filling-in of the atmospheric oxygen bands. Nevertheless, since its first development, its design has been substantially modified. In this new version, a set of 3-channels, equipped with specific interference filters from Alluxa (Santa Rosa, California—US), was used to monitor the spectral profile of the O_2_A absorption band (Fig. 4a). The transmission band of one filter is centered at the minimum of the O_2_ absorption band (in-band), and the other two are placed right before and after the O_2_ absorption feature (out-band). The three filters dedicated to monitoring the O_2_B band in the first version of the instrument were replaced by two narrow bandpass filters in the green (523.8 nm and 566.7 nm) to measure the photochemical reflectance index (PRI). Unfortunately, electronic failures at the campaign's onset precluded signal measurements in the 566 nm channel, and thus PRI could not be calculated. In addition, a third red filter at 672.6 nm and one of the far-red filters used for the O_2_A band profile (770.64 nm) were devoted to determining NDVI. The peak positions, spectral bandwidth (FWHM), and peak transmittance of these filters are listed in Table 1. This new filter set has better transmission and durability than the previous ones. The Airflex objective and the filters are maintained at 40 ± 0.1 °C by a regulated heating system to prevent thermal drifts.

**Table 1:** Peak wavelength (λ_i_), Full Width at Half Maximum (FWHM), and Transmission for the filters used in the Airflex sensor

Airflex sensor filters			
Band	Peak wavelength (nm)	FWHM (nm)	Transmittance
PRI	λ_1_ = 532.8	9.4	95%
PRI	λ_2_ = 566.7	10.2	91%
NDVI	λ_3_ = 672.6	10.8	94%
O_2_A out band	λ_4_ = 757.93	1.13	88%
O_2_A in band	λ_5_ = 760.80	1.02	91%
O_2_A out band / NDVI	λ_6_ = 770.64	1.10	85%

The former Airflex was a cylinder of ≈ 1 m in length; its central part was a hub in front of the objective. The hub contained a set of black-painted baffles to prevent stray light from entering the Airflex objective (Fig. 4b). At the end of this cylinder was the photometer itself (Moya et al. 2006).

### The airflex splitter and the fiber optics

The Airflex hub was removed and replaced by an optical fiber that feeds the light to the objective through a light "splitter" to fit the reduced available space inside the ultralight plane (see Fig. 4b, c). The fiber has a 2 m length and a numerical aperture of 0.37 and 0.94 mm in diameter (SEDI-ATI Fibres Optiques, Courcouronnes, France). The fiber etendue is calculated as the product of the solid acceptance angle by the entrance pupil and is 0.31 mm^2^ rad in our case. This value is greater than the etendue of the native Airflex, which was 0.27 mm^2^ rad. As a fully optimized optical system produces an image with the same etendue as the source, we should provide at least this value. So, we lost a small amount of the collected light that is not accepted by the Airflex etendue.

**Fig. 5:**
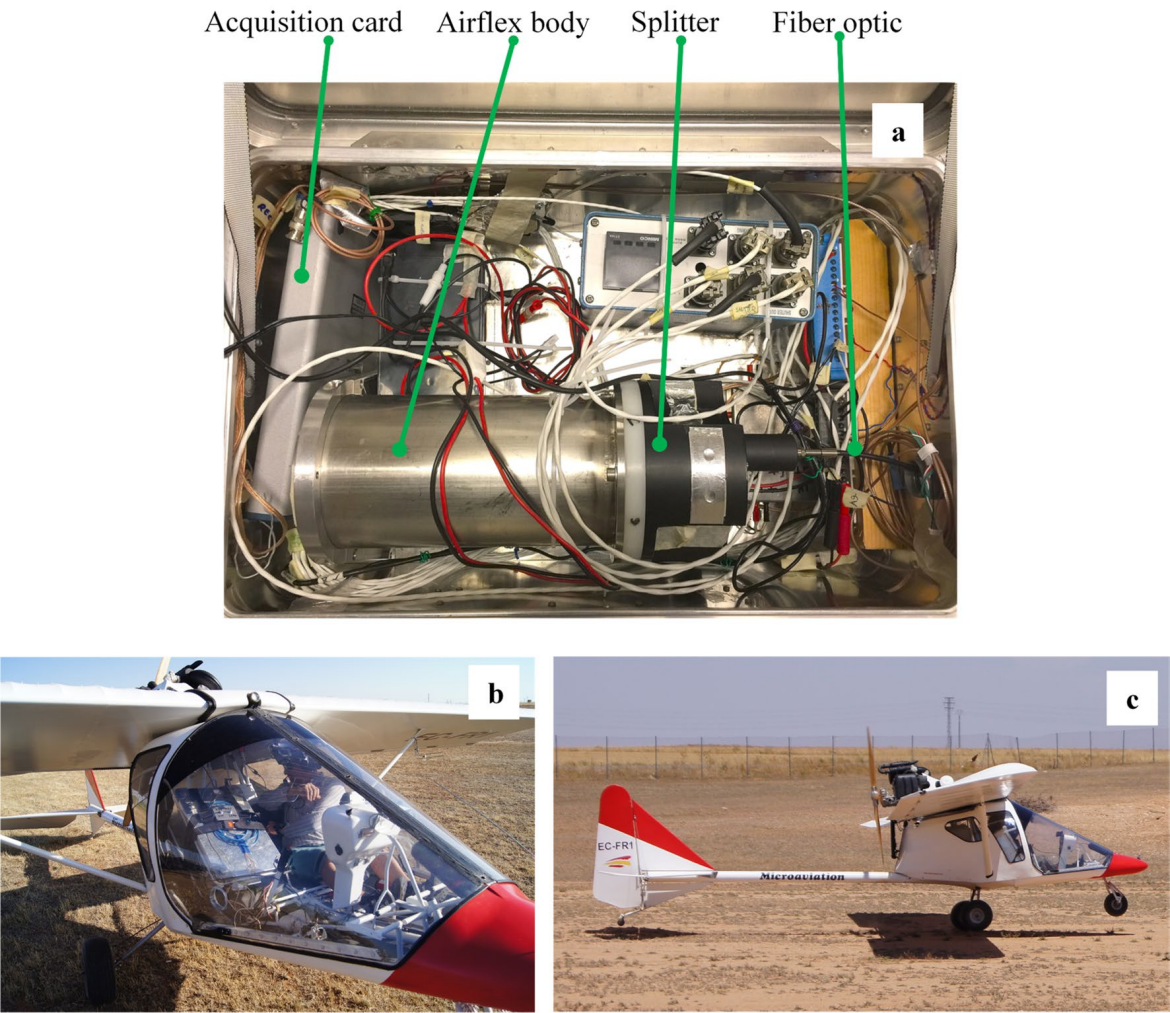
**a** Airflex and associated electronics inside the instrumental box. **b** The Airflex box in the airplane cabin. **c**. The ultralight airplane at take-off

The splitter is a complex piece of machined aluminum that has been specially designed and built in the lab's workshop (Fig. 4c, d). It contains several mirrors, beam splitters, and dichroic filters and divides the flux collected by the optical fiber into six beams corresponding to the six channels described in Table 1. As a result, the optical fiber and splitter system is much more flexible than the original Airflex hub and can fit easily in the cabin of a small airplane, as shown in Fig. 5b.

Each channel signal was measured by a low noise amplified silicon photodiode (HUV 1100 BG, Perkin-Elmer, France). Thanks to an aspheric objective of 25 mm in diameter and 31.25 mm of focal length (Edmund Optics, France), the field of view was set to 3.0 m from an altitude of 100 m. This altitude was maintained more or less constant (± 10 m) during all flights.

Data acquisition was ensured by a data acquisition board (USB NI 6356, National Instruments, France) having eight simultaneous differential inputs with 16 bits encoding capacity. The acquisition software was managed using LabVIEW (National Instruments, France). In addition, a USB monochrome video camera (Chameleon, 1.3 MP CCD, FLIR systems U.S.) was added to record a synchronized image of the field of view context for each Airflex measurement. The video camera was externally triggered by a TTL signal generated by the LabVIEW program. Recording and transferring images were managed by a program developed in C language using libraries of the FlyCapture 2.0 software development kit (SDK) from FLIR Systems that allowed the data acquisition synchronization.

The new version of Airflex with fiber optic, splitter, and a video camera was flown on the 16th (control days), 24th (stress), and 25th (reversion day) of July 2017. During these days, measurements were taken around noon to minimize changes in the atmospheric oxygen absorption band during the flight. The speed was maintained as low as possible within security limits at 25–30 m.s^−1^ (i.e., ≈100 km.h^−1^) at an altitude of approximately 100 m. The ultra-light plane was a Micro Aviation Pulsar III, capable of carrying two persons (Fig. 5c).

The new version of Airflex was radiometrically calibrated just before the airborne campaign with a calibration source (Licor 1800–02, NE, USA) and a second time at the end of the experiment. Both calibrations were in agreement, except for the failure of the PRI channel, which was out of order.

### Canopy reflectance measurements

Canopy reflectance spectra were recorded using a portable fiber optic spectrometer (HR 4000, Ocean Insight, USA). Measurements were taken on the stressed area before starting the water stress period (see Fig. 13 of the appendix). The target size was about 0.7 m in diameter. Measurements on the target were immediately preceded and followed by a similar measurement on a horizontal white-roughened PVC board whose reflectance spectrum was determined in the laboratory against a Spectralon (Labsphere, USA) reflectance standard.

### Chlorophyll fluorescence measurements on the ground

The shape of the fluorescence emission spectrum (Fig. 13 of the appendix) is needed to retrieve the SIF level according to the retrieval method used by Daumard et al. (2010). Therefore, we used the entire sunlight leaf-level emission spectra as a proxy for the canopy level emission, as Fournier et al., (2012) suggested. Before starting the water stress period, measurements were taken on the stressed area. The spectra were acquired with a fluorometer already described by Moya et al. (2006), Rascher et al. (2009), and Daumard et al. (2010), using the sun as the source of excitation. It is based on a portable spectrometer (HR2000 + , Ocean Insight, USA) equipped with a high-pass red filter (RG665, Schott, France) to select wavelengths only corresponding to chlorophyll fluorescence.

The solar radiation was filtered in the illumination system by a low-pass filter (Corning 4.96, 5 mm, Corning, USA), blocking excitation at λ > 600 nm. A plano-convex lens focuses the sun on the leaf to compensate for the light attenuation introduced by the filter and the optics. As a result, measurements were taken at total sunlight excitation. Measurements were performed after a light adaptation period of 10–15 min when a stationary state was reached. Raw emission spectra were corrected for the spectrometer's instrumental response function and the red filter's transmission.

### Statistics

**Fig. 6:**
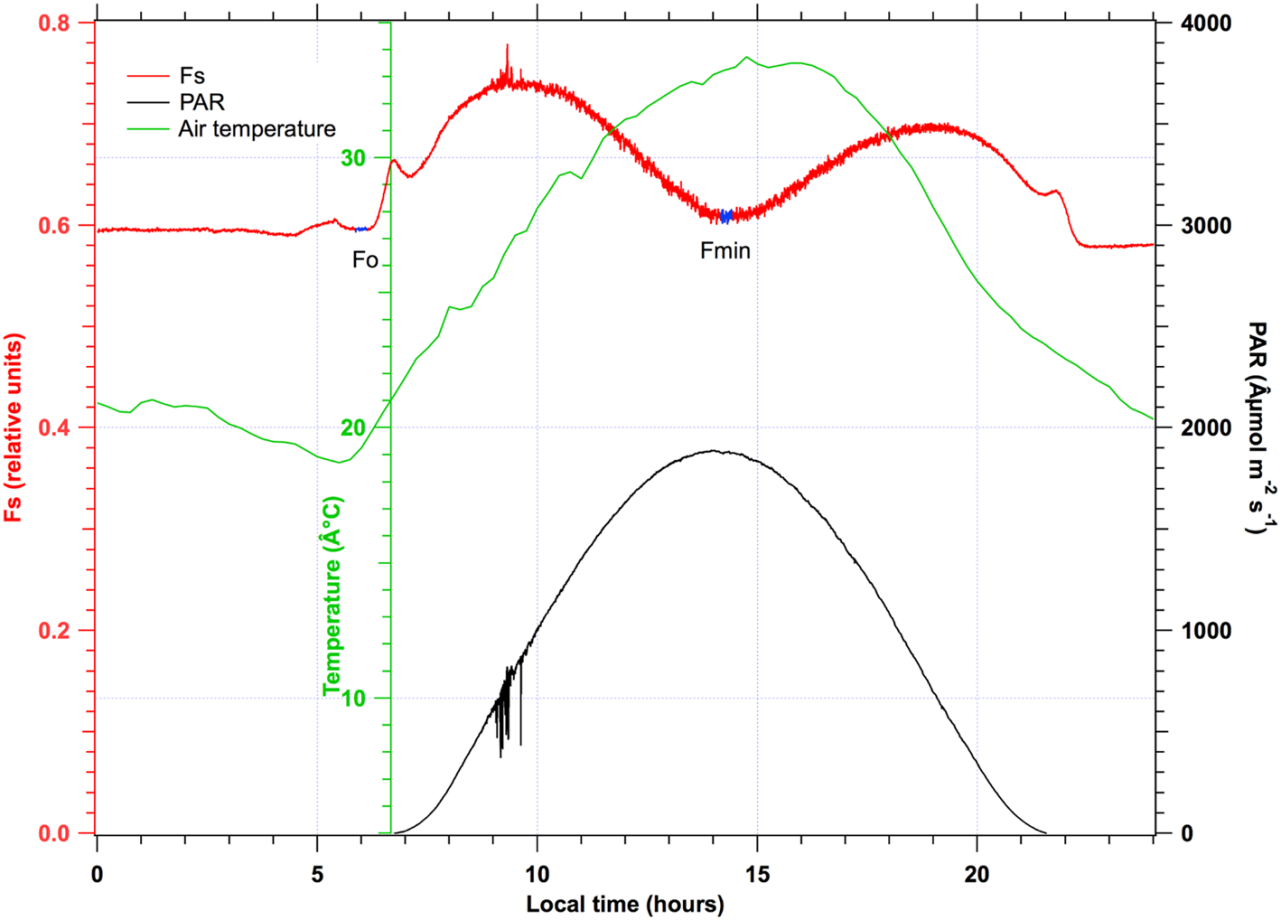
Diurnal time courses of acquired signals during the control day of July 15. Stationary fluorescence is in red. Points in blue on the Fs curve indicate the time interval for the computation of Fo and Fmin (see M&M). In black is the Photosynthetic Active Radiation (PAR). In green is the air temperature measured by the lysimeter meteorological station. Each parameter is associated with a vertical axis of the same color

Mean values of stationary fluorescence level measured by Ledflex were computed at two specific times of the diurnal cycle:

- the fluorescence level in the dark-adapted state (Fo) was derived as the mean of fluorescence in a time interval of 15 min lasting from 05:53 to 06:08 local time. It corresponds to the fluorescence level just before the morning induction curve resulting from the first increase in PAR level.
- the minimum fluorescence level in the day (Fmin) was derived as the mean in a time interval of 15 min around the minimum fluorescence level that occurs around solar noon (Fig. 6).

The standard deviation of Fo and Fmin were also computed on the same sample of data. The number of measuring points included in the computation of each fluorescence level (Fo, Fim) was between 462 and 470, corresponding to a mean sampling rate of raw data of 0.52 Hz.

Linear regression and other statistical analyses were performed using Igor Pro (Wavemetrics, Portland, OR, USA).

## Results

The active fluorometer Ledflex was continuously operated throughout the campaign at about 4 m above ground and monitored the transition of the restricted watering zone from a well-watered situation to stress conditions. The restricted watering phase lasted from July 13 through July 26 (Fig. 6 shows a typical control day, July 15). The stationary fluorescence is in red. In black is the Photosynthetic Active Radiation (PAR) measured with a quantum sensor over the Ledflex instrument (seeFig. 2). The air temperature measured with the meteorological station is green.

**Fig. 7:**
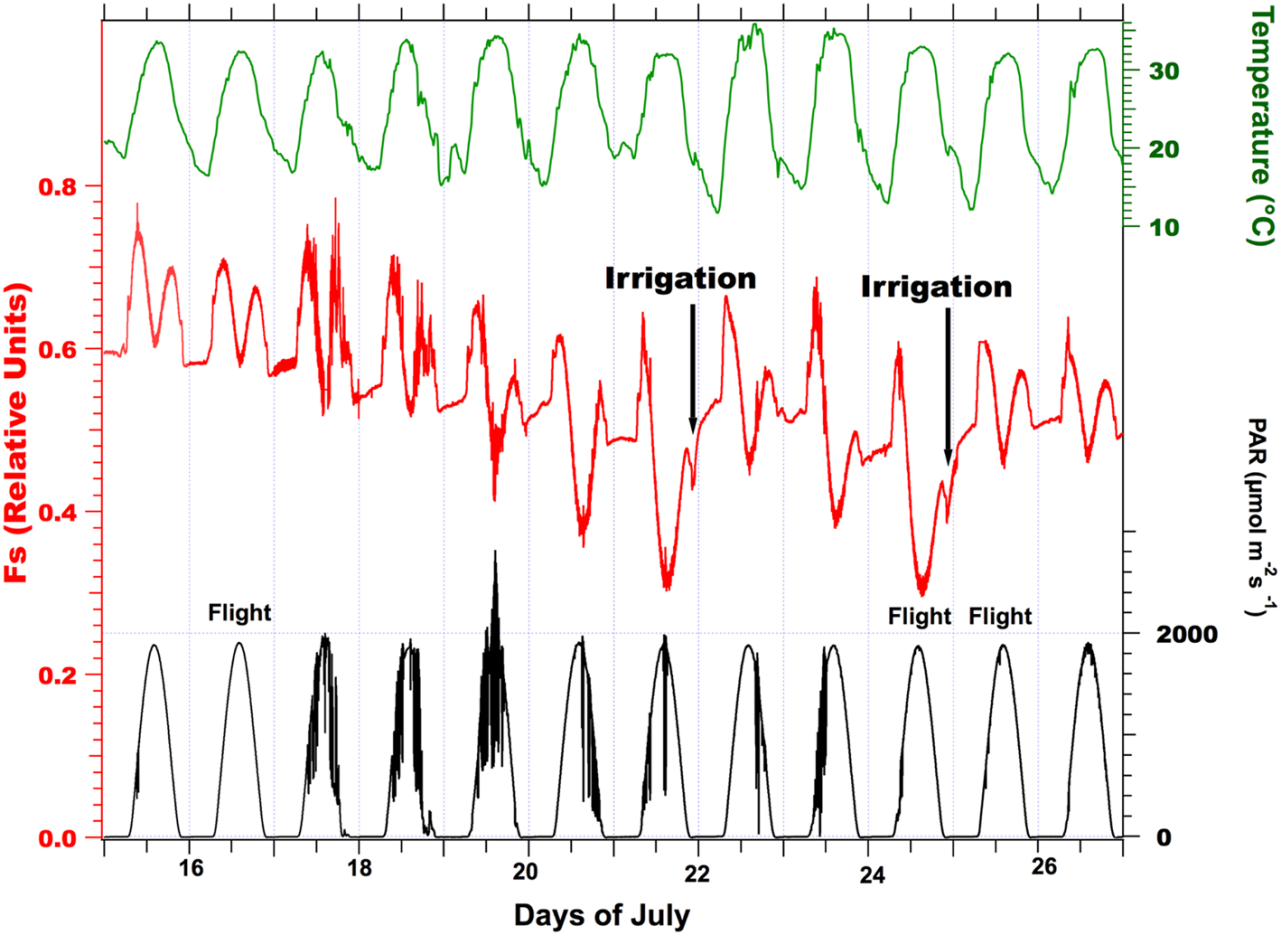
Effect of water stress on stationary fluorescence and recovery after irrigation. From top to bottom: air temperature measured by the meteorological station (green), stationary fluorescence level Fs (red), and PAR (black). The irrigation and airborne observation events are also indicated. Observe the decrease of Fs and the somewhat reproducible diurnal time course of PAR

Figure 7 shows an experiment that starts at midnight and lasts 24 h. Except for some spurious morning variations, the PAR variation describes a very smooth curve close to a cosine curve with a maximum of about 1900 µmol of photons m^−2^ s^−1^. Fs stays almost constant during the night. This constant night level is denoted hereafter as Fo. Fs increases in the morning when PAR increases. A maximum is reached around 9:00 local time with a PAR of 650 µmol of photons m^−2^ s^−1^, then Fs decreases to a minimum (Fmin) slightly above Fo. As light decreases in the afternoon, Fs increases in a somewhat symmetrical way as in the morning but with a smaller amplitude.

**Fig. 8:**
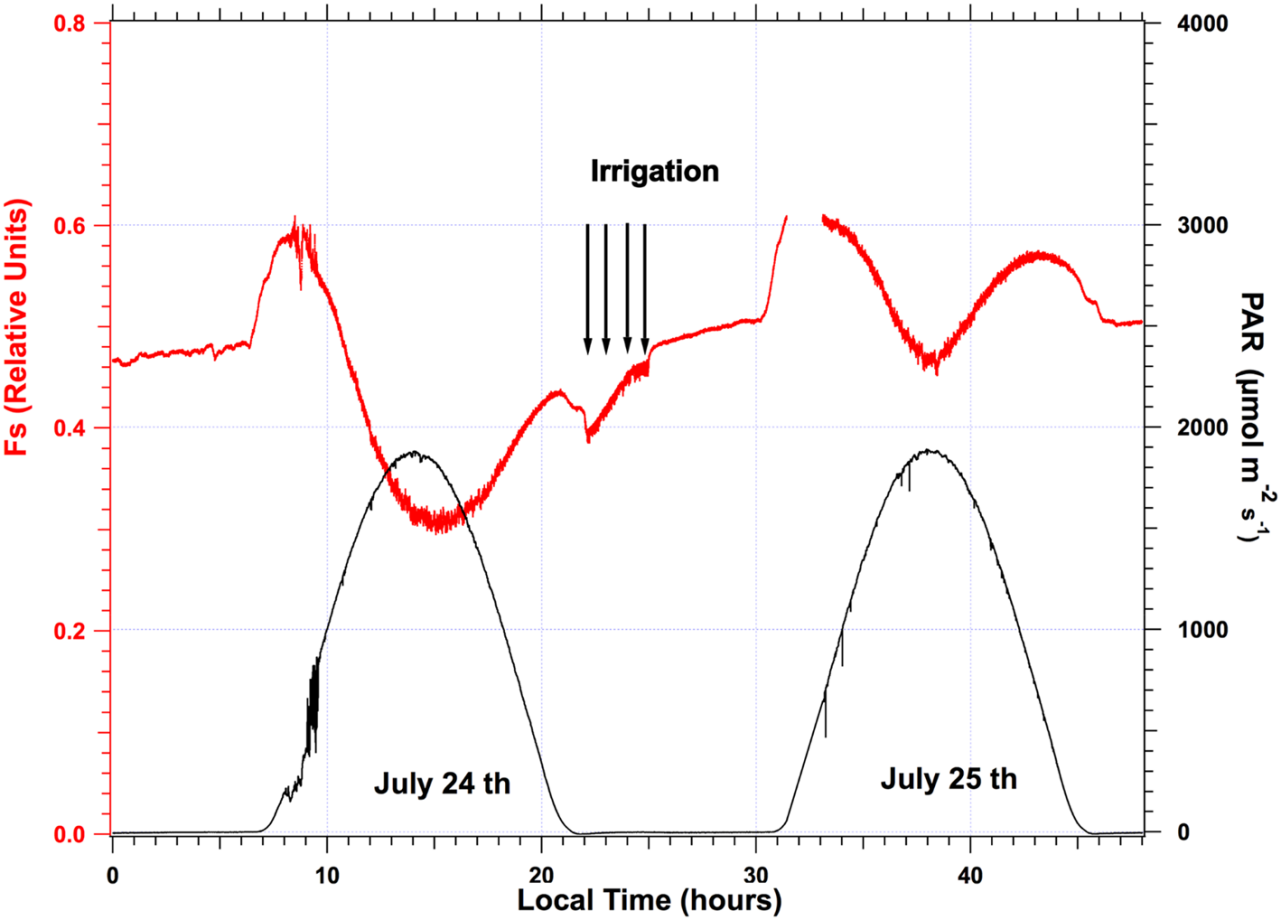
Effect of three hours of irrigation at the end of the water stress period. The minimum fluorescence level (Fs, red) during the day (Fmin) is strongly enhanced, despite similar PAR conditions (black). Observe also the transient decrease of Fs during watering

The irrigation was applied regularly every 2–3 days until July 13, when the interruption of irrigation started for the stressed plot. After July 15, Fs continuously declined in the absence of irrigation or rain until July 21, when watering started again. During this period, sunny conditions were pervasive, as can be seen in the PAR plot of Fig. 7. This drought period induced a significant fluorescence decrease illustrated by the temporal series in which the value of Fs at noon is, at the end of the drought period (July 24), about 53% of its initial value (control day on July 15). On July 21, 1-h night irrigation was implemented to prevent damage to the fescue field. The exact irrigation time can be detected by a decrease in Fs (see Fig. 8). It is interesting to note that just a few hours after watering, the daily cycle of July 22 presents a noticeable Fs increase, showing a pattern very similar to those of July 19. After this temporary increase, Fs decreased again at a similar rate until the night of July 24, when more extended irrigation (3 h) took place. The almost instantaneous effect of watering is better illustrated by comparing the cycles from July 24th and 25th, as shown in Fig. 8. The significant increase of Fs after watering is illustrated by Fmin, which changes from ≈ 0.305 to 0.466 under a similar illumination level. However, despite the irrigation, Fmin stays lower than Fo.

**Fig. 9:**
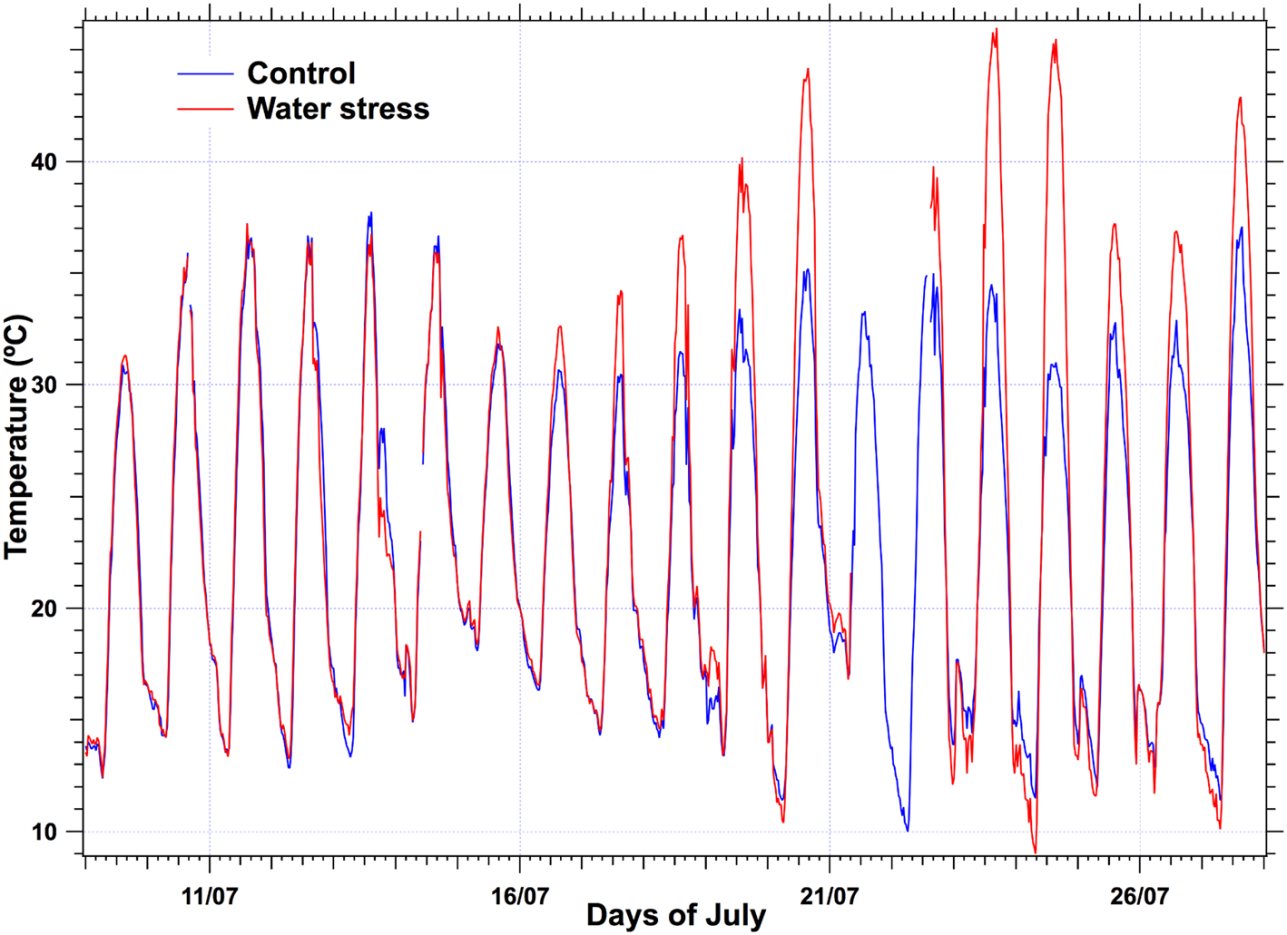
Temporal series of surface temperatures as measured by the Apogee thermometers. While minimal temperatures are unchanged, maximal temperatures of the stressed plot increased more than ten °C during the stress period from July 16 to July 25

The temporal series of surface temperature recorded by the infrared Apogee thermometers situated in both control and stressed parts of the fescue field showed identical measurements until July 16. After this date, the diurnal temperatures of the stressed plot went up to 10 °C above the control. Notwithstanding, night temperatures were identical (Fig. 9).

**Fig. 10:**
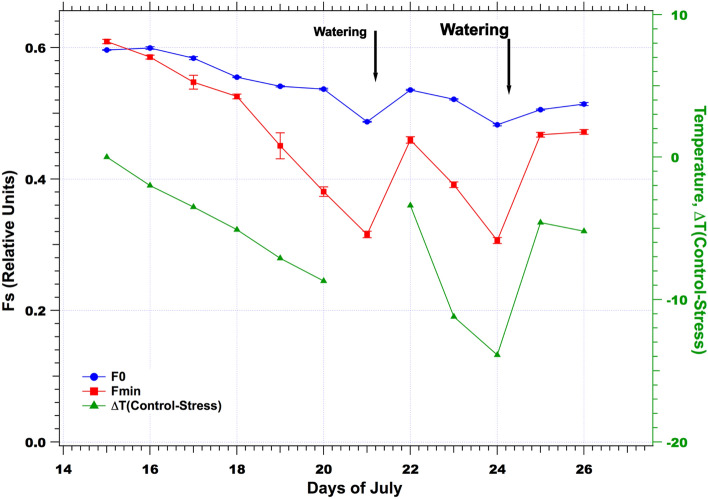
Temporal series of Fo (blue), Fmin (red), and surface temperature difference between control and stressed plots (green) at noon

As shown in Fig. 10, Fo and Fmin decreased concomitantly with the temperature difference between control and stressed plots measured by the IRTs. One may observe the continuous decrease of both Fo and Fmin until July 21 and the partial recovery after the first irrigation, followed by a second monotonous decrease until the second irrigation on July 24. It is worth noting that the surface temperature difference between control and stressed plots followed precisely the same pattern (Fig. 10). Linear regressions between fluorescence data and surface temperature difference result in a coefficient of determination R^2^ = 0.87 and 0.57 for the prediction of ΔT(Control – Stressed) by Fmin and Fo, respectively.

### Airflex data

Airplane data were collected around or just after solar noon, where fluorescence quenching was supposed to be maximum, as shown by the occurrence of Fmin in the Ledflex data (Fig. 7). The plane flew alternatively and several times over empty and fescue fields. As in other works (Moya et al., 2006, 2019; Daumard et al. 2015), the atmospheric oxygen band depth at 760 nm (D_760_) was characterized by the ratio of the out-band signal F_757.93_ (at 757.93 nm) to the in-band signal F_760.8_ (at 760.8 nm):

$${\text{D}}_{{{76}0}} = {\text{ F}}_{{{757}.{93}}} /{\text{ F}}_{{{76}0.{8}}}$$

D_760_ at the sensor level was in the order of 3.5 over bare soil (without vegetation), and we took this value as the reference to calculate the vegetation fluorescence fluxes (Moya et al. 2006; Daumard et al. 2015). The contribution of the fluorescence to the vegetation radiance led to a decrease of D_760_ from bare soil to vegetation covered fields. Over the fescue parcel, this decrease was about 0.13–0.15, corresponding to a filling-in of the atmospheric O_2_ band (not shown).

**Fig. 11:**
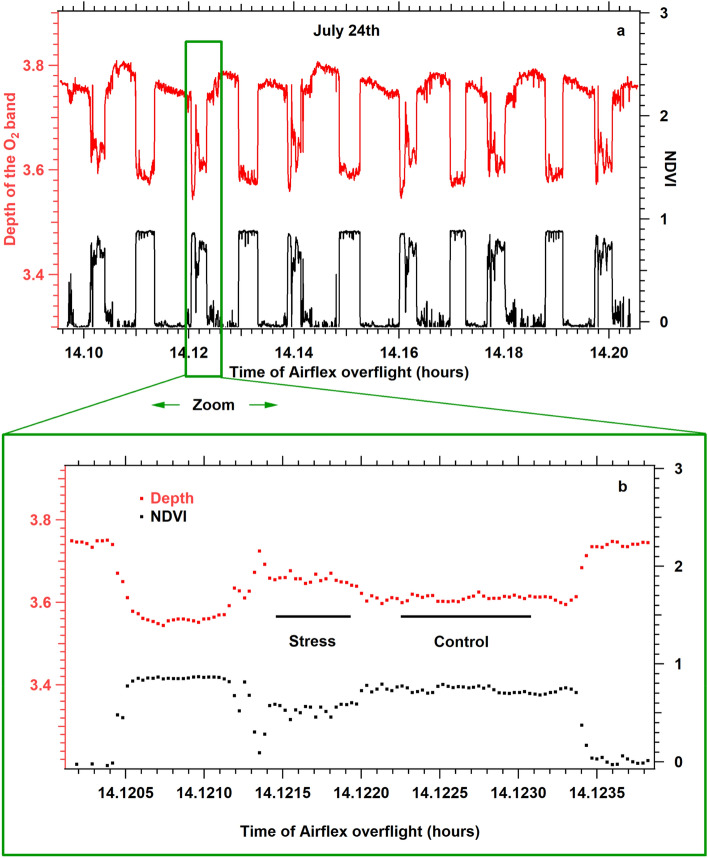
**a** Reproducibility of depth measurements (red) and NDVI (black). **b** Enlarged detail of a consecutive measurement of both stress and control plots

Depth measurements are illustrated in Fig. 11a, representing a 10 min flight over a succession of empty and green fields. Two curves directly calculated from measured radiances are presented: the band depth D_760_ (in red) and the normalized difference vegetation index (NDVI) (Bannari et al. 1995) in black. One may appreciate both curves' good reproducibility despite the pilot's difficulty reproducing exact overflights. In our case, NDVI was defined according to Airflex filters:

$${\text{NDVI }} = \, \left( {{\text{F}}_{{{77}0.{64}}} - {\text{ F}}_{{{672}.{6}}} } \right) \, / \, \left( {{\text{F}}_{{{77}0.{64}}} + {\text{ F}}_{{{672}.{6}}} } \right)$$

where: F_672.6_ and F_770.64_ represented the radiance in the 672.6 and 770.64 nm channels, respectively.

NDVI was almost zero for empty fields, whereas vegetation parcels (maize) had an NDVI of about 0.9, and fescue fields had a lower NDVI, between 0.6 and 0.8. Figure 11b shows an example of the identified zones thanks to the images of the context taken for each measuring point (not shown) and from which depths and NDVI were extracted.

In Table 2, the main results of the flight campaign are reported, including NDVI's and depths for fescue under stressed and watered conditions. The reproducibility of the flights can be judged by the depth measured over bare soils, which changed by only 1.85% during the nine days of the airborne campaign. However, it was difficult to get a constant depth level over bare soils, as illustrated in Fig. 11a for July 24 (stressed case), and this is also true for the other days. Several reasons can be invoked to explain this difficulty, including loops made by the plane to repeat the same trajectory, the wind always present in the afternoon, and the weakness of the navigation tools that equipped this tiny plane. For the control plot, NDVI increased continuously from 0.683 to 0.790 (13.5%), probably due to growth during the nine days of the airborne measurements. At the same time, stressed plot NDVI initiated the campaign with a lower value (0.651), decreased during the drought period reaching 0.575 (July 24), and seemed to rise upon irrigation (0.663) on July 25 (see Table 2).

**Table 2:** Summary of flight data

Date of flight	NDVI control	NDVI stress	D760 bare field	D760 control	D760 stress	D760 (Stress)—D760 (Control)	F_yield *10–3
July 16th 2017	0.683	0.651	3.455	3.315	3.329	0.014	5.57 ± 0.64
July 24th 2017	0.764	0.575	3.52	3.369	3.414	0.045	4.42 ± 0.47
July 25th 2017	0.790	0.663	3.511	3.382	3.414	0.032	4.46 ± 0.38

Apparent fluorescence yield (F_yield) was computed over the stressed plot where Ledflex was situated

The O_2_ band depth for the control plot showed a variability (1.98%) similar to the band depth variation of the bare soils. The depth of the O_2_ band increased on July 24 compared to July 16. An increase in the depths is expected if the amount of fluorescence decreases. In all cases, D_760_(stress) > D_760_(control) indicates a decrease in the stressed plot fluorescence compared to the control (Table 2).

By chance, both control and stressed plots were measured consecutively with 2—3 s of delay, ensuring identical measuring conditions that were not guaranteed when comparing different days. For this reason, the differences between depths of stressed minus control plots were emphasized (Table 2).

**Fig. 12:**
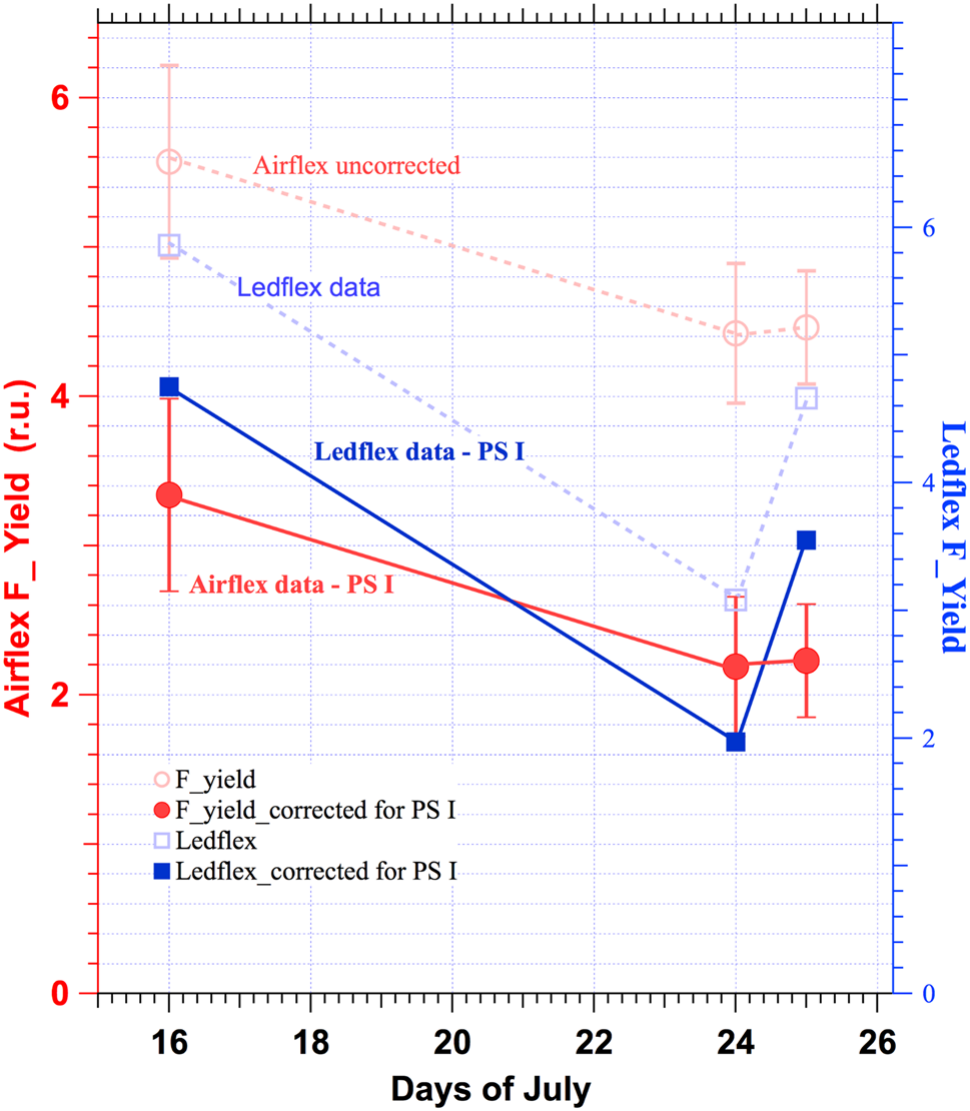
Red empty circles, dashed lines, and left axis correspond to the total fluorescence yield measured by Airflex. Red solid circles: after subtracting PS I fluorescence contribution (≈ 40%). Right axis empty blue squares correspond to Ledflex fluorescence data—solid blue squares: Fluorescence yield after subtracting PS I fluorescence emission (≈ 19%)

**Fig. 13:**
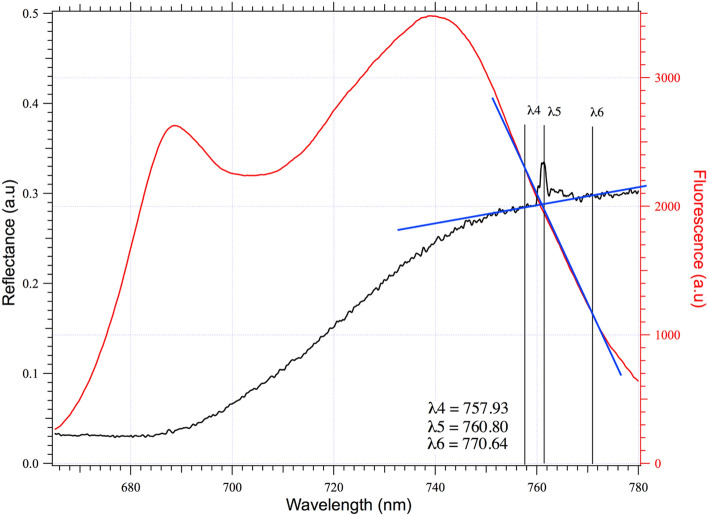
Reflectance spectrum (black) and fluorescence emission spectrum (red) of a well-watered fes-cue crop. Blue lines indicate the linear models of reflectance and fluorescence emission used for fluorescence retrieval in the O2A absorption band. Chlorophyll concentration ≈ 20 SPAD units

The fluorescence flux at the ground was computed using the method of Daumard et al. (2010), which combines a linear model of the reflectance spectrum in the vicinity of 760 nm and the shape of the fluorescence emission spectrum measured on the ground as described in the methods (see Appendix) to decouple reflectance and fluorescence emission in the vegetation radiance. The vegetation radiance at three wavelengths (one in-band at 760.8 nm and two out-bands at 757.93 and 770.64 nm) and the same data from bare soil allowed us to calculate the fluorescence flux at 760.8 nm using the equations of Daumard et al. (2010). The Modtran 4 model was used to correct the atmospheric absorption along the path from the vegetation to the sensor (≈100 m) in the airborne signals (Daumard et al., 2007). Results of fluorescence flux were divided by the actual Photosynthetic Active Radiation at the time of measurements to be converted into apparent fluorescence yields (F_yield) and are shown in the last column of Table 2 and Fig. 12.

$${\text{F}}\_{\text{yield }} = {\text{ F }}/{\text{ PAR}}$$

Apparent fluorescence yields were computed only over the stressed plot as measurements with Ledflex were done at this location.

## Discussion

### Ground measurements

Until July 2017, Ledflex was mainly used with potted plants to control irrigation conditions better. For this reason, we took advantage of having a field campaign in the hot conditions of mid-summer prevailing in the center of Spain (Barrax). The results in Fig. 6 were similar to those shown in Moya et al. (2019) for control conditions on potted pea plants. It can be interpreted as follows (Flexas et al. 2000): photochemical quenching (qP) primarily determines the actual fluorescence level when the stomata are open. A light intensity increase modifies the equilibrium of the electron transport chain in the direction of a reduction, which is accompanied by an increase in ChF. After approximately 9.30 am, the light intensity increases, and the plant needs another mechanism to cope with the increase of light. A non-radiative (heat) dissipation of the excess absorbed energy mechanism occurs at the LHCII antennae (NPQ) level that also involves the violaxanthin cycle, inducing a decrease of Fs. Reciprocally, Fs increases when the NPQ relaxes after a light intensity decrease in the afternoon, reproducing an Fs cycle similar to the morning but with slightly lower amplitude because some NPQ persists. Therefore, it will need the night to relax.

Figure 8 (July 24th) presents the situation of the same fescue field after several days of water stress. Again, one may observe the same steps for Fs qualitatively except that the maximum in the morning is reached one hour before, and under a lower light intensity, the minimum at noon is strongly decreased, and Fs always stays lower than Fo.

Other similar results have been found in the literature. For example, in a similar experiment, Rosema et al. (1998) used a target formed by poplar trees grown in pots in a growth cabinet with glass walls inside a greenhouse. An Nd-Yag laser providing pulses of 10 mJ of 10 ns length at 532 nm was used for excitation. The laser illuminated an area of 60 cm in diameter at 12 m. During a five-day water stress experiment, the diurnal cycle showed a dip at noon that developed and became lower than Fo when drought progressed. Indeed, inside a greenhouse with low radiation (< 400 μmol m^−2^ s^−1^), the water stress signature was evidenced at the canopy level.

Cerovic et al. (1996) used a modified PAM 101 (Walz, Effeltrich, Germany) to monitor Fs at a distance of up to 1 m on an attached leaf. The authors monitored several species submitted to drought, including maize, sugar beet, and kalanchoë. After six days of withholding watering on maize, Fs decreased at noon to a value lower than Fo. Although in this experiment, the light intensity was limited to less than 350 μmol m^−2^ s^−1^ for technical reasons, these results align with the data presented here.

Flexas et al. (2000) also studied water stress's effect on an attached vine plant leaf during a campaign of 17 days. The authors developed a new fluorometer based on a laser diode for measuring at a distance both Fs and Fm' through the window of an LI-6400 gas analyzer. They also evidenced the M shape of the Fs diurnal cycle with a minimum at solar noon. Under stress conditions, the evening branch was much lower than the morning one, and the minimum was lower than Fo, in agreement with what is shown in Fig. 8 of the present paper.

Bright light conditions prevailed in an outdoors vineyard work presented by Lopez (2015). They used a laser-diode μLidar, developed at LMD (Laboratoire de Météorologie Dynamique, Paris), which was able to measure Fs from a few meters distance over a plant section containing several leaves. Fieldwork was conducted during the summer, for 45 days, at Barrax, in the South of Spain. Fs was continuously measured from well-watered conditions (stomatal conductance Gs = 0.18 mol H_2_O m^−2^ s^−1^) to stress conditions (Gs = 0.05 mol H_2_O m^−2^ s^−1^). During this long period of good weather, neither the chlorophyll content nor the reflectance was modified. The authors observed a progressive decrease of Fs at noon, which dropped below Fo at the end of the treatment. Notably, 12 h after re-watering, a diurnal cycle similar to control plants was obtained.

Nevertheless, fescue results showed that Fmin is close to or just below the Fo level even under well-watered conditions at noon, whereas in the control pea experiment shown in Moya et al. (2019), Fmin ≈ 1.16 Fo under similar conditions. Up to now, only a few species, including peas, sweet potato, mint, and grapevine, have been tested, and we always found Fmin > Fo under well-watered conditions. At least three reasons can be evoked to explain the low Fmin value observed on the fescue crop: i.) The weekly mowing strongly reduces the height of the crop and tends to reduce the shade within the fescue canopy and increases the illumination, ii.) the high irradiance conditions prevailing near the summer solstice in the South of Spain, and iii.) the high average temperature associated with this continuous high irradiance. The co-occurrence of these three conditions may produce a substantial decrease in Fs, even lower than the level registered at noon.

To conclude, with ground-based measurements in the fescue experiment, it is evident that both Ledflex and temperature difference data measured at noon (with the help of a control plot) demonstrated a high sensitivity to detect reversible water stress. Figure 10 is a good summary of measurements at ground level. Then, why not use just temperature rather than fluorescence to detect water stress, as temperature measurements are much easier and cheaper to install? The answer could be that we need a reference (control) field to calculate a temperature difference, whereas a single stressed plot is enough for Ledflex fluorescence measurements as we can compare the minimum fluorescence level reached during the day (Fmin) with the Fo level observed overnight.

### Airborne measurements

Data shown in Fig. 12 and Table 2, evidence the capacity of passive measurements to detect a decrease in apparent fluorescence yield during a water stress period. More precisely, the fluorescence yield for the control observed on July 24 was ≈ 0.79 ± 0.126 of the value measured on July 16 (Table 2). A slightly recovering signal can be observed on July 25 after three hours of watering. Although having only three points, the overall pattern of Fig. 12 can be compared with data changes shown in Fig. 10.

On the other hand, the Fmin value measured with Ledflex on July 24 (maximum stress) was 0.53 of the value reached on July 16. Therefore, we should conclude that the Airflex and Ledflex data do not match quantitatively. In order to get an explanation for these differences, let us emphasize some essential differences in the chlorophyll excitation between the Ledflex and Airflex instruments.

Active methods like Ledflex use traditionally narrow band excitation often produced by lasers or LEDs. The energy is usually concentrated in a narrow spectral domain, which helps to isolate fluorescence from excitation spectrally. For instance, Ledflex excites fluorescence at 470 ± 5 nm with a narrow bandwidth of ≈ 20 nm. This wavelength is well absorbed by photosystem II (PS II) and by carotenoids (Louis et al. 2006) and corresponds to a minimum absorption of photosystem I (PS I) (see Fig. 2 of Laisk et al. 2014). This figure allowed us to compare the blue excitation of PS I that coincides with depression around 470 nm with other more efficient wavelengths to excite PS I. Assuming the contribution of PS I excitation to be 35% at longer wavelengths (Pfündel 1998; Agati et al. 2000; Franck et al. 2002), the lower absorption of 470 nm leads to a lower excitation of PS I. We concluded with an overall contribution of 19% of PS I for Ledflex fluorescence measurements.

Airflex uses a passive method to detect sun-induced chlorophyll fluorescence in the O_2_A absorption band at 760 nm in a narrow band of ≈ 1 nm, excited by the whole solar spectrum. Considering the several works already cited concerning the participation of PS I in the far-red fluorescence emission, we guess it is also the case at 760 nm (Franck et al. 2002). It is worth noting that F760 also benefits from a unique excitation by wavelengths between 690 and 750 nm that is predominantly absorbed by PS I, compared to PS II. Laisk et al. (2013) stated that the excitation spectra of PS I electron transport are strongly favored at wavelengths greater than 690 nm compared to PS II. This work estimates an extra excitation of ≈ 15% in favor of PS I. This extra excitation does not exist in active methods, especially in the case of Ledflex. In other words, PS I emission can account for approximately 35 × 1.15 ≈ 40% of the emission at 760 nm. On the control day (July 16), we have Fs = Fo at noon (see Fig. 7). We then assume a constant PS I emission of ≈ 40% of Fs superimposed to the PS II emission.

After nine days of withholding watering, Airflex data shows a decrease in the apparent fluorescence yield at noon to 0.79 compared to the control day on July 16 (see Table 2). This decrease can be due to a decrease in PS II fluorescence or PS I fluorescence, or both. It has been shown (Dau 1994; Trissl 1997) that open or closed PS I reaction centers are equally efficient in trapping the excitation energy from the antennae; hence, there is no variable PS I fluorescence. As a result, we tentatively attribute the fluorescence decrease of 0.79 to the quenching of PS II, and we supposed that the PS I contribution remained unchanged during the nine days of water stress.

As both instruments were differently affected by the contribution of PS I fluorescence emission that we did not measure, the comparison becomes delicate. Therefore, we presented in Fig. 12 an attempt to compare both results after removing the supposed PS I contribution that accounted, as described, for ≈ 40% of Fmin for Airflex and ≈ 19% of Fmin for Ledflex. Although these values are speculative, considering the error bars, they seem realistic and compatible with a decrease of ≈ 53%, as shown by Ledflex active data.

## Conclusions

Although active and passive chlorophyll fluorescence measurements are qualitatively in agreement, the accuracy and sensitivity of the active method are much better. It also provides additional information as it can detect fluorescence at night and under low light. Under solar excitation, passive measurements using the filling-in of the atmospheric oxygen band at 760 nm do not seem to be an appropriate wavelength to detect water stress due to the significant contribution of the PS I fluorescence, which is always superimposed with PS II emission, and rather under-documented. A more effective wavelength should be to use the O_2_B band at 687 nm, where the contribution of PS I is marginal. However, due to several factors, fluorescence detection in the O_2_B band is considered more complex than in the O_2_A band. One of these factors is the non-linear shape of the reflectance spectrum over the O_2_B band, which implies using not three but at least four wavelengths to correctly describe the curvature of the reflectance spectrum with multi-channels photoradiometers like Airflex (Daumard et al. 2012).

However, alternative retrieval methods, such as the spectral fitting method (SFM) (Cogliati et al. 2019), take advantage of the comprehensive information contained in high spectral resolution radiance spectra to retrieve both the red and far-red emissions of chlorophyll fluorescence. Such a method will be implemented in the data processing chain of the future FLuorescence EXplorer (FLEX) space mission of the European Space Agency (ESA) to provide the full fluorescence spectrum, including the red band (Drusch et al. 2017). Our results, considered from the perspective of future fluorescence space missions, open new possibilities for water stress detection from space.

## Data Availability

The data supporting this study's findings are available in the Dataverse CGIAR repository at Active in situ and passive airborne fluorescence measurements for water stress detection on a fescue field. https://doi.org/10.21223/7QY6KU

## Appendix

### Fluorescence retrieval method

Here, we briefly describe the retrieval method used to recover solar-induced fluorescence from airborne radiance measurements obtained with the Airflex sensor. More detailed information can be obtained from Daumard et al. (2010, 2012, 2015).

At the ground level, the vegetation radiance in the vicinity of the O_2_ absorption band can be described as:

1 $${\text{L}}(0,\lambda ) \, = \rho (\lambda ) \;{\text{I}}(0,\lambda ) \, + {\text{ F}}(0,\lambda ),\;\lambda \in \left\{ {\lambda {4}, \, \lambda {5}, \, \lambda {6}} \right\}$$

where ρ(λ) is the reflectance factor, defined as the ratio of the energy flux reflected by the target to the energy flux reflected by a white Lambertian reference board in the same configuration, I(0, λ) is the solar irradiance on the target expressed in radiance units (i.e., the solar irradiance divided by π) and F(0, λ) is the fluorescence radiance generated at the surface by the I(0, λ) excitation. In Eq. (1), L(0, λ), ρ(λ), I(0, λ) and F(0, λ) represent their respective spectral values integrated over the spectral bandwidth of the Airflex filters and λ is the peak wavelength of the corresponding filter (see Table 1 and Fig. 13).

It is worth noting that because ρ(λ), and F (0, λ) are both unknown, Eq. (1) cannot be solved, in a general case, whatever the number of wavelengths considered. As a consequence, additional information is necessary to retrieve fluorescence flux. To solve Eq. (1), we employed the method described by Daumard et al. (2010) and further applied it in Fournier et al. (2012) and Daumard et al. (2012). In short, the method uses complementary information obtained on the ground to model ρ(λ), F(0, λ) and I(0, λ) spectra.

*The solar excitation model.* The solar spectrum I(0, λ) was measured at the ground level at the time of the flight using a white PVC board calibrated against the Spectralon board reference, as stated in the methods section. Here we assumed I(0, λ) to be constant during the flight period.

*The reflectance model.* Under sunlight excitation, only apparent reflectance r(λ) can be measured, which may contain some fluorescence contribution.

$${\text{r}}(\lambda ) \, = {\text{ L}}(0,\lambda ) \, /{\text{ I}}(0,\lambda ) \, = \rho (\lambda ) \, + {\text{ F}}(0,\lambda ) \, /{\text{ I}}(0,\lambda )$$

The Chl fluorescence contribution to the radiance of the vegetation is known to be < 2% of the continuum radiance at 760 nm (Moya et al. 2004). We decided to neglect such a small contribution. This leads us to choose a linear model for ρ(λ) in the vicinity of the O_2_A band as in similar studies (Daumard et al. (2010, 2012), Fournier et al. (2012).

Figure 13 shows apparent reflectance at the ground measured as stated in the methods section. Although reflectance spectra are relatively smooth, one may observe a prominent peak at wavelength λ5. This peak is produced by the fluorescence contribution to the vegetation radiance and showed a maximum effect at the bottom of the O_2_ absorption bands. This peak is not observed when measuring the reflectance of empty fields (not shown). As suggested in Fig. 13, we took a linear model.

2 $$\rho (\lambda ) \, = {\text{ a}}\lambda + {\text{ b}},\; \lambda \in \left\{ {\lambda {4}, \, \lambda {5}, \, \lambda {6}} \right\}$$

where a and b are the coefficients of the reflectance model.

Figure 13 also shows the emission spectrum of the fescue taken before the water stress period. One may observe that the slope of the fluorescence spectrum is remarkably constant between λ4 and λ6. This allows us to model the F(0, λ) by a linear function that we characterize by two constant parameters:

$${\text{A}}(\lambda {4}) \, = {\text{ F}}(0,\lambda {4}) \, /{\text{ F}}(0,\lambda {5}) \, = { 1}.{\text{15 and}}$$

$${\text{A}}(\lambda {6}) \, = {\text{ F}}(0,\;\lambda {6}) \, /{\text{ F}}(0,\;\lambda {5}) \, = \, 0.{6}$$

By definition A(λ5) = 1.

The system (1), which corresponds to ground parameters, can be rewritten as follows:

3 $${\text{L }}(0,\lambda {4}) \, = \rho (\lambda {4}){\text{ I}}(0,\;\lambda {4}) \, + {\text{ F }}(0,\;\lambda {5}){\text{ A}}(\lambda {4}),$$

$${\text{L }}(0,\lambda {5}) \, = \rho (\lambda {5}){\text{ I}}(0,\;\lambda {5}) \, + {\text{ F }}(0,\;\lambda {5}),$$

$${\text{L }}(0,\lambda {6}) \, = \rho (\lambda {6}){\text{ I}}(0,\;\lambda {6}) \, + {\text{ F }}(0,\;\lambda {5}){\text{ A}}(\lambda {6}),$$

ρ(λ4) = a λ4 + b,

ρ(λ5) = a λ5 + b,

ρ(λ6) = a λ6 + b

As measurements were taken at the airplane altitude, we should introduce some corrections for the contribution of the air mass between the sensor and the ground. The target radiance at altitude h (L(h, λ)) can be expressed in terms of ground irradiance I(0, λ) and fluorescence F (0, λ5), taking into account absorption and scattering of reflected solar radiation and fluorescence by the atmospheric column between ground and sensor.

The Modtran4 model was used to calculate the mean attenuation Ts(λi) over the filter spectral bandwidth of the solar radiance flux reflected by the target. As the filter bandwidth is narrow (about 1 nm, see Table 1), the solar radiance at the airplane level can be formulated as:

4 $${\text{Is}}({\text{h}},\lambda {\text{i}}) \, = {\text{ Ts}}({\text{h}},\lambda {\text{i}}) \;{\text{I}}(0,\lambda {\text{i}}),\;\lambda {\text{i}} \in \left\{ {\lambda {4}, \, \lambda {5}, \, \lambda {6}} \right\}$$

It can be observed that only inband radiance was significantly modified. Indeed higher altitudes affected all the channels, but they are out of the scope of the present work. Calculating the attenuation undergone by the fluorescence emission generated from the surface F(0, λi) in the path from the ground to the sensor is also necessary. The transmission of the air column for the fluorescence Tf(h, λ) is calculated using the Modtran4 model in the transmission mode between the ground level and the airplane altitudes:

5 $${\text{F}}({\text{h}},\lambda {\text{i}}) \, = {\text{ Tf}}({\text{h}},\lambda {\text{i}}){\text{ F}}(0,\lambda {\text{i}}), \lambda {\text{i}} \in \left\{ {\lambda {4}, \, \lambda {5}, \, \lambda {6}} \right\}$$

The attenuation of fluorescence flux induced by atmospheric corrections was 12%

**Table 3:** Ai coefficients, transmission factors of solar radiation, and fluorescence along the path from ground to sensor were calculated according to Daumard et al. (2015)

Wavelength channel	Airflex filter peak wavelength (nm)	Form factors of the fluorescence emission spectrum (A(λ))	Transmittance of solar radiation (Ts) calculated with Modtran4	Transmittance of fluorescence (Tf) calculated with Modtran4
λ4	757.93	1.15	0.99	0.989
λ5	760.80	1	0.983	0.882
λ6	770.64	0.6	1	0.989

^3^). Finally, by introducing Eqs. (4) and (5) into the system (3), we obtain the following system of linear Eqs. (6) relating solar irradiance and Chl fluorescence at the ground to the measured onboard radiances at the airplane altitude:

6 $$\left\{ {\begin{array}{*{20}c} {{\text{L }}({\text{h}},\lambda {4}) = } & {\rho (\lambda {4}){\text{ I}}(0,\lambda {4}){\text{Ts}}({\text{h}},\lambda {4}) \, + {\text{ F}}(0,\lambda {5})\;{\text{A}}(\lambda {4}){\text{ Tf}}({\text{h}},\lambda {4})} \\ {{\text{L}}({\text{h}},\lambda {5}) \, = } & {\rho (\lambda {5}){\text{ I}}(0,\lambda {5}){\text{Ts}}({\text{h}},\lambda {5}) \, + {\text{ F}}(0,\lambda {5}){\text{Tf}}({\text{h}},\lambda {5})\;\;\;\;\;\;\;\;\;\;\;\;\;\;} \\ {{\text{L}}({\text{h}},\lambda {6}) \, = } & {\rho (\lambda {6}){\text{ I}}(0,\lambda {6}){\text{Ts}}({\text{h}},\lambda {6}) \, + {\text{ F}}(0,\lambda {5}){\text{A}}(\lambda {6}){\text{ Tf}}({\text{h}},\lambda {6})\;\;\;} \\ {\rho (\lambda {4})\;\;\;\;\; = } & {{\text{ a}}\lambda {4} + {\text{b }}} \\ {\rho (\lambda {5})\;\;\;\;\; = } & {{\text{ a}}\lambda {5} + {\text{b }}} \\ {\rho (\lambda {6})\;\;\;\;\; = } & {{\text{ a}}\lambda {6} + {\text{b }}} \\ \end{array} } \right.$$

By eliminating ρ(λi) λi$\in \{\uplambda 4,\uplambda 5,\uplambda 6\}$, a, and b coefficients, the system of Eqs. (6) has a unique solution for F(0, λ5). The solution can be easily found using Mathematica™ software:

$${\text{F}}(0,\lambda {5}){\text{ N}}\left( {\lambda {5}} \right) \, /{\text{ D}}\left( {\lambda {5}} \right)$$

where:

$${\text{N}}\left( {\lambda {5}} \right) \, = {\text{ I}}\left( {0, \, \lambda {5}} \right){\text{ I}}\left( {0, \, \lambda {6}} \right){\text{ L}}\left( {{\text{h}}, \, \lambda {4}} \right)\left( { \, \lambda {5}{-} \, \lambda {6}} \right){\text{Ts}}\left( {{\text{H}}, \, \lambda {5}} \right){\text{ Ts}}\left( {{\text{h}}, \, \lambda {6}} \right) \, + {\text{ I}}\left( {0, \, \lambda {4}} \right){\text{Ts}}\left( {{\text{h}}, \, \lambda {4}} \right) \, \left[ {{\text{I}}\left( {0, \, \lambda {5}} \right){\text{ L}}\left( {{\text{h}}, \, \lambda {6}} \right) \, \left( {\lambda {4}{-}\lambda {5}} \right){\text{Ts}}\left( {{\text{h}}, \, \lambda {5}} \right) \, + {\text{ I}}\left( {0, \, \lambda {6}} \right){\text{L}}\left( {{\text{h}}, \, \lambda {5}} \right) \, \left( {\lambda {6}{-} \, \lambda {4}} \right){\text{Ts}}\left( {{\text{h}}, \, \lambda {6}} \right)} \right]$$

and:

$${\text{D}}(\lambda {5})\, = \,{\text{A}}(\lambda {4}){\text{ I}}(0,\lambda {5}){\text{ I}}(0,\lambda {6})(\lambda {5}{-}\lambda {6}){\text{ Ts}}({\text{h}},\lambda {5}){\text{ Ts}}({\text{h}},\lambda {6}){\text{ Tf}}({\text{h}},\lambda {4}) + \,{\text{I}}(0,\lambda {4}){\text{ Ts}}(0,\lambda {4}) \, [{\text{I}}(0,\lambda {6}) \, [(\lambda {6}{-}\lambda {4}){\text{Ts}}({\text{h}},\lambda {6}){\text{ Tf}}({\text{h}},\lambda {5}) \, + {\text{ A}}\left( {\lambda {6}} \right){\text{ I}}\left( {0, \, \lambda {5}} \right) \, \left( {\lambda {4}{-}\lambda {5}} \right){\text{Ts}}\left( {{\text{h}}, \, \lambda {5}} \right){\text{Tf}}\left( {{\text{h}}, \, \lambda {6}} \right)]]$$

## Abbreviations

SIF: Solar-induced fluorescence
PAR: Photosynthetically active radiation
O_2_A: Oxygen A absorption band
O_2_B: Oxygen B absorption band
D760: The atmospheric oxygen band depth at 760 nm
F_yield: Apparent fluorescence yield at 760 nm
Fs: Stationary fluorescence yield
Fo: Minimum dark-adapted fluorescence yield
Fmin: Minimum of stationary fluorescence yield at around solar noon
Tair: Air temperature measured by the meteorological station
NDVI: The normalized difference vegetation index
PS I: Photosystem I
PS II: Photosystem II
